# Nanobodies As Novel Agents for Targeting Angiogenesis in Solid Cancers

**DOI:** 10.3389/fimmu.2017.01746

**Published:** 2017-12-08

**Authors:** Roghaye Arezumand, Abbas Alibakhshi, Javad Ranjbari, Ali Ramazani, Serge Muyldermans

**Affiliations:** ^1^Department of Biotechnology and Molecular Science, School of Medicine, North Khorasan University of Medical Sciences, Bojnourd, Iran; ^2^Department of Biotechnology, School of Advanced Technologies in Medicine, Shahid Beheshti University of Medical Sciences, Tehran, Iran; ^3^Cancer Gene Therapy Research Center, Zanjan University of Medical Sciences, Zanjan, Iran; ^4^Cellular and Molecular Immunology, Vrije Universiteit Brussel, Brussels, Belgium

**Keywords:** angiogenesis, nanobody, monoclonal antibody, cancer therapy, vascular endothelial growth factor family

## Abstract

Solid cancers are dependent on angiogenesis for sustenance. The FDA approval of Bevacizumab in 2004 inspired many scientists to develop more inhibitors of angiogenesis. Although several monoclonal antibodies (mAbs) are being administered to successfully combat various pathologies, the complexity and large size of mAbs seem to narrow the therapeutic applications. To improve the performance of cancer therapeutics, including those blocking tumor angiogenesis, attractive strategies such as miniaturization of the antibodies have been introduced. Nanobodies (Nbs), small single-domain antigen-binding antibody fragments, are becoming promising therapeutic and diagnostic proteins in oncology due to their favorable unique structural and functional properties. This review focuses on the potential and state of the art of Nbs to inhibit the angiogenic process for therapy and the use of labeled Nbs for non-invasive *in vivo* imaging of the tumors.

## Introduction

Chemotherapy, radiotherapy, and surgery are routine methods to eradicate tumor tissues; however, nowadays more efficient and less harmful methods are in sight. Carefully selected monoclonal antibodies (mAbs) have been shown to exert strong suppression of tumor growth. These effects are provoked by different strategies, such as a direct targeting of malignant cells, delivering cytotoxic moieties, modifying the host immune response, and retargeting the cellular immunity toward malignant cells (1, 2). Since proliferating cancer cells induce and form new blood vessels to meet their needs for nutrients, inhibition of blood vessel formation seems to be an attractive option for cancer therapy (3).

During the past decades, administration of mAbs as cancer therapeutics has increased steadily, and currently, approximately 350 mAbs have entered clinical trial programs and over 70 intact antibodies or fragments thereof received approval from FDA or EMEA for clinical applications (4). Although, the mouse hybridoma technique, developed in 1975, pioneered the identification of mAbs of defined specificity, this first generation of mAbs failed to fulfill its promises to produce therapeutics, mainly because the rodent origin of mAbs provoked severe immune responses in humans. Chimeric, humanized, and fully human mAbs were developed to remedy these immunogenicity problems and nearly all currently marketed antibodies belong to one of these types (5). Despite these improvements, the full potential of mAbs remains curtailed due to their large size, necessity to be produced in a multimeric format, high production and purification costs and their poor diffusion within tissues and the solid tumor (6). Intact functional mAbs are complex, glycosylated proteins with a molecular mass of about 150 kDa. Moreover to be used as therapeutic proteins, they have to be produced and purified in large quantities under GMP conditions. In addition, the vast majority of the administered mAbs usually remains in the bloodstream, and despite having a high specificity for a particular tumor associated antigen, they fail to reach and to associate with their target outside the blood compartment (7).

**Figure 1:**
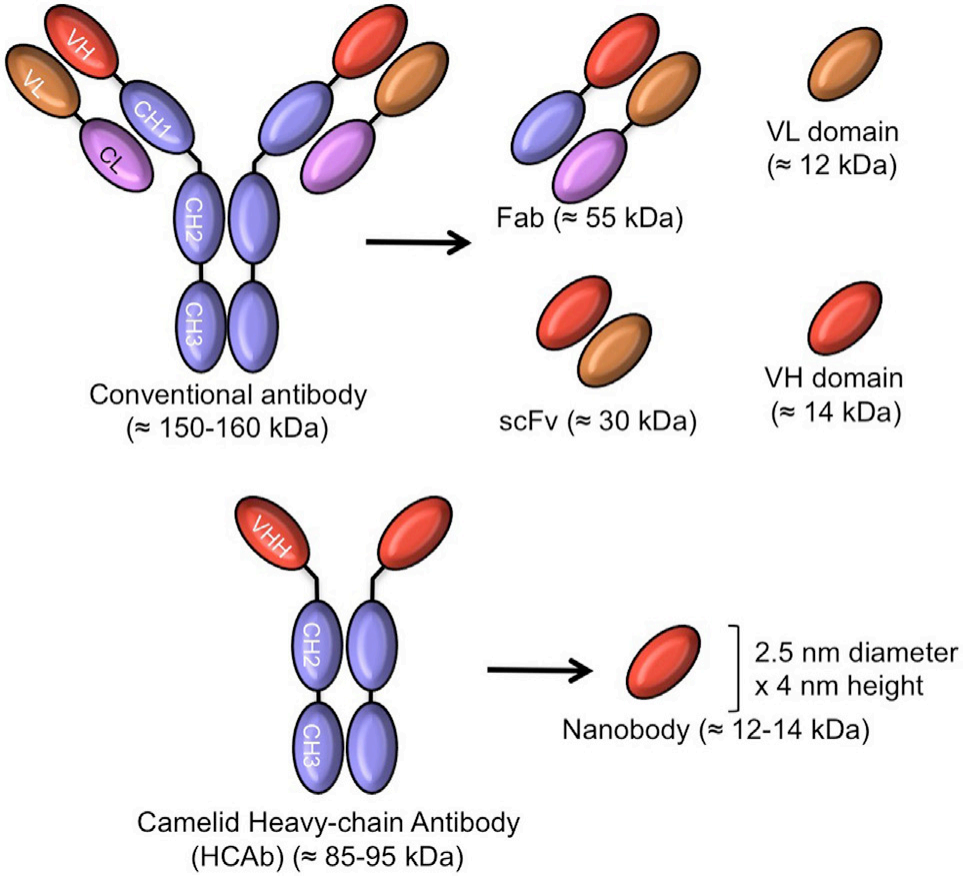
Schematic representation of intact antibody and antibody-derived fragments of conventional and camelid heavy chain-only antibodies (HCAb). The molecular mass of all molecules is given in parentheses.

The miniaturization of mAbs to smaller antigen-binding fragments (Figure 1) avoids many of the above shortcomings as these products overcome

- the necessity of using complex expression systems,
- the poor diffusion within the solid tumor, and
- the nonspecific Fc-dependent immunologic responses.

In contrast to whole antibodies, microbial expression systems manage to produce functional antigen-binding fragments in high yields. These smaller antigen-binding fragments are produced successfully at an industrial scale in microbial systems (bacteria, yeasts, and fungi), providing access to a faster and larger production of a safer drug at a lower cost. So, antibody fragments including the antigen-binding fragment (Fab ~55 kDa), the single chain variable Fragment (scFv: 26–30 kDa), and single-domain antibody fragments (sdAb ~12–14 kDa) have been expressed from bacteria and yeasts for therapeutic purposes and are indeed being evaluated in clinical trials (8, 9).

The search for smaller antibody fragments eventually led to the development of engineered sdAbs consisting of the variable fragment (about 110 amino acids) of heavy or light chain immunoglobulin polypeptides. These sdAbs from human origin as engineered originally at Domantis (Cambridge, UK, now part of GlaxoSmithKline) may overcome immunogenicity issues and could be administered orally, by inhalation or topically in a gel or cream (10).

Remarkably, an alternative to human immunoglobulin to generate sdAbs was proposed from the serendipitous discovery of heavy chain-only antibodies (HCAb) in sera of camelids. These are naturally occurring, functional antibodies, devoid of light chains, and without the first constant domain, the CH1 (11). They recognize the antigen by virtue of the *v*ariable domain of the *h*eavy chain of *h*eavy chain-only antibodies (known as VHH). The recombinant VHH was later on also referred to as nanobody (Nb), because of its dimensions in the low nanometer scale (MW: 12–14 kDa) (12). The amino acid sequence of VHH (as occurring in camelids) is close to sequences of family 3 of human VH and up until now Nbs are not generally immunogenic in patients participating in clinical trial projects (13).

The differences between VH from classical antibodies and VHH from HCAbs in the framework region 2 (FR2) and in the length of the CDRs (complementary determining region) are well established (14). Large hydrophobic amino acids in FR2 of the VH domain that associate with the VL (variable light chain), are substituted in VHHs by smaller and/or more hydrophilic amino acids. These mutations increase the solubility and stability of autonomous VHHs in absence of partner VL domains (14). The hypervariable region that overlaps with CDR1, is extended by four more amino acids toward the N terminal end and this region in a VHH is probably involved directly or indirectly in antigen recognition. Likewise, the CDR3 is on average longer in a VHH than in a VH and part of it can form a protruding loop contacting grooves or concave epitope architectures on the surface of the antigen (15–17). These longer CDR1 and CDR3 loops are regularly connected through an interloop disulfide bond (in camels or dromedaries), which might further contribute favorably to the stability of the domain under stringent conditions such as elevated temperature or pH extremes (18).

Among the main properties of Nbs, we note their

- small size (1/10 of intact conventional antibody),
- high degree of sequence identity with human VH,
- high expression in microbial hosts,
- high stability and solubility, and
- high specificity and affinity for their cognate antigen.

Each of these characteristics leads to a number of beneficial outcomes (14) and their robustness even allows gut passage of orally taken Nbs to reduce the morbidity of rotavirus infected animals (19).

The small size and monomeric behavior of Nbs facilitates their gene manipulation (Table 1) and assists their penetration into tumor tissues where a prevalent high pressure of the interstitial space prevents the transport of larger molecules (whole antibodies) (16). Although the monomeric Nbs are rapidly cleared from the blood *via* the kidneys (half-life of around 30 min), nonetheless, a high tumor accumulation can be reached (20). The conjugation of Nbs with nuclides or dyes generates tracers for usage in non-invasive, *in vivo* imaging of tumors for diagnosis or to monitor the therapeutic treatment (21). Furthermore, the monovalent Nb can be easily manipulated to form bivalent, multivalent, bispecific, or bi-paratopic constructs. The fusion of a Nb to another Nb with specificity to albumin increases its half-life blood retention from less than 30 min to 2–3 days (22).

**Table 1:** Characteristics of nanobodies (Nbs) (23–25).

Nb	The recombinant form of the variable antigen-binding domain of heavy chain-only antibodies (HCAbs) from camelids

Main sources	HCAbs expressed from peripheral blood lymphocytes of camelids (bactrian camel, dromedary, llama, and vicugna)

Size	2.5 nm diameter, 4 nm height (molecular mass 12–15 kDa)

Structure	A single monomeric, variable immunoglobulin domain

Function	Binds specifically and with high affinity to its cognate antigen

Preferred production technique	Immunization of camelids to raise a HCAbs immune response; cloning of VHH repertoire from peripheral blood lymphocytes; retrieval of antigen-specific Nbs after phage display (or other display methods)

Application areas	Therapeutic applications: selective toxin neutralizing or tumor targeting (e.g., targeted radionuclide therapy); Diagnosis: non-invasive *in vivo* imaging; antigen capturing agent in micro-arrays and biosensors; Research: affinity chromatography, crystallization chaperones, drug discovery, intracellular expression, and target tracing, elimination, modulating, relocation, degradation,….

Biochemical properties	High expression yields in microorganisms; nano- to picomolar affinities; recognition of unique epitopes; generally non-immunogenic; facile gene manipulation

Biophysical properties	Stability usually higher than conventional antibodies; high solubility; rapid blood clearance; fast tissue penetration, short half-life in blood due to renal clearance and absence of Fc/FcR interaction

Disadvantages	Small size may cause problems in parenteral applications; lack of effector function-mediated effects; increased frequency of dosing for systemic applications

The natural source of Nbs, in addition to their unique properties have attracted a lot of attention and many research groups are currently developing new Nbs as candidates for therapeutic and diagnostic applications (Table 1). Nb ALX0061 and Nb ALX00171 against interleukin 6 receptor and respiratory syncytial virus (RSV) for treatment of rheumatoid arthritis and RSV infection might be on the market soon (8). In addition, several Nb-based therapeutic agents against TNF-α, IL17A (26), VEGF/angiopoietin-2 (Ang-2) (27), CXCR1, CXCR2 (28), vWF (29), RANKL (30) applicable in autoimmune disease, malignant disease, inflammation, hematopoitic disorders, and bone disorder, respectively, are at various stages of clinical trials.

The aforementioned beneficial properties support Nbs as potent agents in targeting a wide variety of disease-related antigens, especially those related to cancer. At present there are many more active projects on the identification and development of new Nbs against cancer specific antigens.

## Nb Generation Techniques

### Phage Display

Phage display is powerful technique to retrieve binders against various targets from a large and diverse library (31). This technology applied for Nbs after immunizing a camelid turns out to be relatively fast and efficient, certainly since the animal can be immunized simultaneously with multiple antigens and a hyper immunization scheme can be shortened to about 6 weeks. The phage display vectors have been adapted for a straightforward cloning of Nbs amplified by RT-PCR. To improve the transformation efficiency, phagemid vectors, such as pCOM3 and pHEN series have been designed and used in combination with M13K07, R408, or VCSM13 helper phages to produce monovalent displayed Nbs at the tip of the virions (32). The immune Nb libraries are unique as they give access to the intact, affinity-matured, antigen-binding fragments. This means that a relative small library is sufficiently adequate to retrieve potent antigen-binders. Immune or naïve scFv libraries and even synthetic man-made scaffold libraries require much larger libraries to retrieve specific binders of high affinity although synthetic or naïve phage display system have remediated some limitations of hybridoma or immune libraries (e.g., in the case where the target is a weak immunogen) (33). However, the success of retrieving good binders is correlated with the size and diversity of the library. Fresh blood and quality of mRNA and cDNA preparations are very crucial to construct a high quality library and to ensure the identification of potent binders (34).

### Other Techniques

Apart from phage display, which remains the first choice because of its robustness, alternative selection techniques for Nbs have also been successfully applied, including ribosome or mRNA display, yeast or bacterial surface display, and even bacterial two-hybrid screenings (35). The multivalent display of Nbs in yeast or bacterial display systems in combination with fluorescent activated cell sorter (FACS) selection allows a rapid identification of the very best Nbs within the library. Conversely, the acellular ribosome and mRNA display techniques clearly avoid the transformation step into an *E. coli* host. In addition, the reverse transcriptase and PCR amplification steps after each round of selection, might introduce minor sequence variations that could contribute to the identification of stronger binders (36).

## Angiogenesis in Cancer

**Figure 2:**
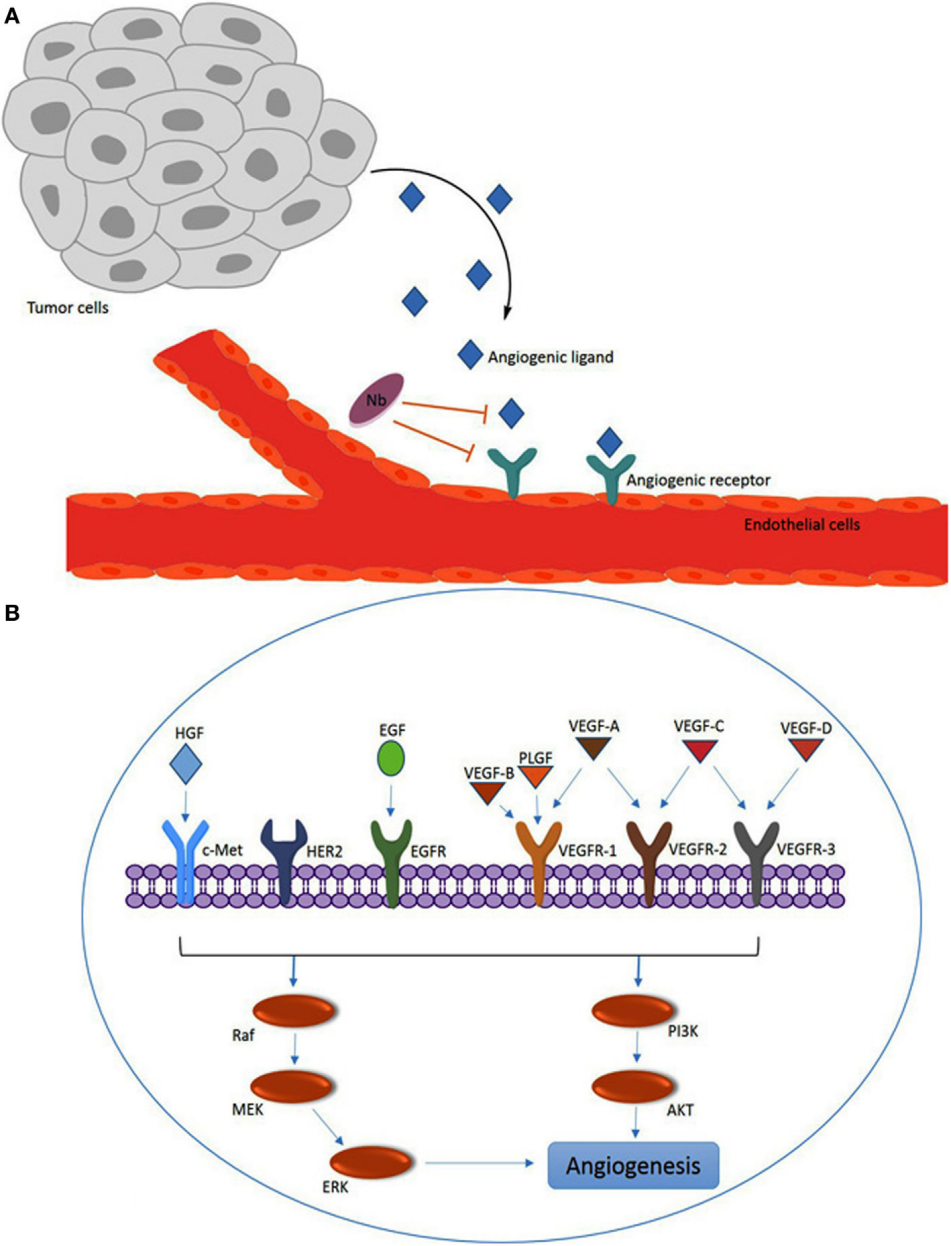
Overview of angiogenesis induced by the vascular endothelial growth factor (VEGF) family members and other angiogenesis factors. **(A)** Angiogenic ligands (e.g., VEGF) are released by tumors and captured by ligand receptors [e.g., VEGF receptor (VEGFR)] on endothelial cells. The nanobodies (Nbs) with specificity for the ligand or the receptor can interfere with for example the VEGF–VEGFR interaction by steric hindrance upon binding to VEGF or VEGFR. **(B)** Overview of major VEGF/VEGFR family members and other angiogenesis factors [c-Met, HER2 and epidermal growth factor receptor (EGFR); and hepatocyte growth factor (HGF) and EGF] that are involved in intracellular signaling *via* the PI3K or Raf pathways to promote angiogenesis.

Angiogenesis is the physiologic pathway whereby new blood vessels are formed from existing vessels. These new vessels are induced by various stimulators such as hypoxia, vessel damage, or angiogenesis growth factors that act as environmental triggers (37). This process needs to be controlled under strict conditions and each disturbance in its balance might cause pathologic distress such as tumorigenesis. Tumor angiogenesis is one of the main properties of cancer cells whereby new blood vessels are formed in the vicinity of the tumor so that tumor cells are supplied with the required oxygen and nutrients. Therefore, upregulation of angiogenesis factors stimulates tumor growth and metastasis. Indeed, some of the angiogenic modulators like members of the vascular endothelial growth factor (VEGF) family and the VEGF receptor (VEGFR) family have a direct role in both the pysiological and the pathological conditions (38) (Figure 2).

In the VEGF/VEGFR signaling pathway, the ligands, including VEGF-A, VEGF-B, VEGF-C, VEGF-D, and placental growth factor (PLGF), interact with membrane bound tyrosine kinase receptors VEGFR-1 (FLT-1), VEGFR-2 (FLK-1/KDR), and VEGFR-3 (FLT4) (Figure 2). VEGFs also bind to particular co-receptors, including neurophilin NRP-1 and NRP-2. The association of VEGF-A (known as VEGF) to VEGFR-2 has been discovered to be a key mediator of angiogenesis. VEGF-A, which is expressed in many human tumors, triggers a number of intracellular signaling cascades in endothelial cells leading to formation and enhancement of tumor microvasculature (39). A variety of factors like AKT, Raf, P13K, MEK, and ERK may be involved in the molecular mechanism of the intracellular signaling pathways of angiogenesis (40).

Furthermore, several studies revealed that tumor tissues express additional factors, such as cancer-associated antigens, that have indirect effects on angiogenesis. Tyrosine kinase inhibitors or mAbs, targeting these angiogenic factors are currently used in the clinic. Despite their strong inhibitory potential of angiogenesis, they offer only limited success in treating cancer patients due to the defense mechanisms of the tumor to escape and to resist the anti-angiogenesis therapy, for example by overexpressing other angiogenesis factors (41).

In the following sections, we will discuss in detail the Nbs that target the angiogenesis factors.

## Major Angiogenesis Targets for Nbs in Cancer

Tumor angiogenesis involves a complex network of interactions. It has been demonstrated that some of the transmembrane proteins, such as tyrosine kinase receptors, are one of the best options for Nb targeting. Here, the prevention of ligand association on the tyrosine kinase receptor by Nbs and avoiding the intracellular cascade signaling is the main objective. Some receptors have several extracellular domains [e.g., epidermal growth factor receptor (EGFR), HER2, c-Met] and thus expose multiple potential epitopes for Nb recognition. Possibly, targeting these epitopes might lead to subsequent internalization of the associated molecule inside the cell, which might be an effective route to transport a drug, toxin, nuclide, or any other harmful substance inside cancer cells. Alternatively, Nbs against the ligand of the transmembrane receptor is also a feasible strategy, especially when the signaling pathway is activated by only one ligand. The effectiveness of this approach was exemplified with Nbs against hepatocyte growth factor (HGF) that activate c-Met, but fail to interfere with the EGFR signaling as this receptor is activated by multiple ligands (12).

The high potential of Nbs as magic bullets that can interfere with angiogenesis are expected to bridge easily the gap from bench to clinic. The targeting of angiogenesis with Nbs has been explored to reach various outcomes. For therapeutic objectives, a clear inhibition of tumor growth and metastasis is required, so that Nbs against the receptor or against the ligand can be employed. For diagnosis and monitoring the treatment follow up, the primary target will be the tumor receptor. For these purposes, the Nb itself should likely be manipulated to improve its potential. These modifications include (i) increasing the valency of Nbs *via* designing bifunctional, bispecific, or bi-paratopic constructs and (ii) increasing the half-life of Nbs either by increasing their hydrodynamic volume (e.g., PEGylation) or by hooking on an abundant compound with long half-life (e.g., decorating the therapeutic Nb directly with albumin or tethering a Nb against serum albumin to the therapeutic Nb). Currently, there are several Nbs that reached different clinical stages and most of these are multivalent Nbs, such as Caplacizumab (28), Vobarilizumab (42), ALX0171 (43), Ozoralizumab (44), and ALX0761 (45).

Vascular endothelial growth factor, because of its strong effect on angiogenesis stimulation, is the main target for development of strategies in anti-angiogenesis therapy. Bevacizumab (Avastin, Genentech, San Francisco, CA, USA), a humanized anti-VEGF-A mAb and potent anti-angiogenesis agent has been approved since 2004, along with other chemotherapeutic drugs (46). Apart from tumor therapy, the VEGF antibody and its antibody fragments have been used to combat other angiogenic disorders, such as age-related macular degeneration (AMD). Aflibercept ((Eleya), Regeneron and Sanofi Aventis, Bridgewater, NJ, USA) is a fusion protein consisting of the VEGF-binding regions of the extracellular domains of human VEGFR-1 or VEGFR-2 fused to the Fc regions of human immunoglobulin G1. This hybrid molecule could block VEGF-A, B, and PLGF in wet AMD and diabetic macular edema and is also being investigated for retinal vein occlusion (46).

Vascular endothelial growth factor is also a major target for development of domain antibodies. Recently, a VEGF dual domain antibody has been reported that seems to be more effective than Avastin or Aflibercept (47). Several studies indicated favorable targeting properties for molecules that combine high affinity and a small molecular size (48).

Following another strategy, Ablynx developed a tri-specific humanized Nb for targeting angiogenesis. In their complex, a Nb against VEGF, a second Nb against Ang-2 and a third Nb against serum albumin for half-life extension are combined. The VEGF and Ang-2 are cross talking and the VEGF upregulates expression of Ang-2. The dual targeting Nb (BI1836880) developed by Ablynx and Boehringer Ingelheim inhibits the VEGF and Tie-2 (the Ang-2 receptor) signaling and prevents the proliferation of endothelial cells. In different *in vivo* models, this Nb construct seems to be superior in efficacy in comparison to Avastin (49).

In one of our studies, we could select VEGF-binding Nbs with specificity for VEGF-121, an isoform of VEGF-A. This Nb inhibits proliferation and tube formation of human umbilical vein endothelial cells (HUVEC) (50). Farajpour et al. developed a Nb against VEGF-165, this binder not only blocked interaction of VEGF with its receptor in cell ELISA but could also prevent significantly the proliferation of HUVEC in a dose-dependent manner (51). Ebrahimzadeh et al. also developed a Nb (VA12) against VEGF, which exhibited high affinity (3 nM) and stability along with significant anti-angiogenesis potential in a chorioallantoic membrane (CAM) assay (52). The CAM assay is an *in vivo* angiogenesis model of fertilized chicken eggs, used for studying the neovascularation (38).

The VEGFR-2, type II of transmembrane tyrosine kinase receptor, expressed on endothelial cells and on circulating bone marrow-derived endothelial progenitor cells, is a key receptor in tumorigenesis. The importance of VEGFR-2 signaling in tumor angiogenesis suggests that targeting of this receptor would be a useful therapeutic strategy to inhibit angiogenesis and tumor growth. Ghavamipour et al. developed a set of high-affinity Nbs (*K*_D_ down to 0.6 nM) against a conformational epitope corresponding to the VEGF-binding domain of VEGFR-2. To increase the chances to retrieve Nbs with this specificity, a combinatorial screening strategy was applied employing a competition phage ELISA panning (53).

In 2012, Behdani et al. reported the identification of an Nb against VEGFR-2 after phage display and panning on immobilized extra cellular domain of VEGFR-2 (54). VEGFR-2, in contrary to VEGFR-1 is switched on in pathologic conditions such as tumorigenesis. In this study, the Nb could inhibit tube formation of HUVEC cells. The Nb was used to develop pseudo-lentiviral vectors for potential transductional targeting of tumor vasculature (55). In another study, Ma et al. selected a high quality Nb against VEGFR-2 domain 3 (VEGFR-2 D3) after panning with antigen in solution. The affinity, as measured by surface plasmon resonance (SPR) indicated a moderate *K*_D_ of 49 ± 1.8 nM for VEGFR-2 domain 3. Nevertheless, this Nb could inhibit the growth and tube formation of HUVEC cells (56).

Placental growth factor is a member of the VEGFs family. This factor is responsible for physiologic angiogenesis but it is also overexpressed in many cancers. Multiple studies have been reporting the anti-angiogenesis effect of PLGF targeting by applying mAbs (57, 58), gene inactivation methods, and antagonist peptides (59). Studies have shown how this factor and its receptor (VEGFR-1) are upregulated in tumor tissues. The mAb against PLGF had inhibitory effects on growth and metastasis in different tumor tissues. This mAb could enhance the effect of chemotherapy agents (57). Currently, a fully human mAb against PLGF called TB-403 entered clinical trials. The results of a phase I dose-escalation study of this humanized anti-PLGF mAb (TB-403, ThromboGenics/BioInvent) plus Bevacizumab in patients with advanced solid tumors were reported. The toxicity profile of this mAb plus Bevacizumab showed promising results (60). Bevacizumab is the first approved angiogenesis inhibitor which has been used for the treatment of metastatic colorectal cancer by inhibiting VEGF-A, one of the other members of VEGF family (61). Recently, our group reported the successful development of a Nb against PLGF. This Nb inhibited the proliferation, migration, and invasion of endothelial and breast cancer. The results of the CAM assay demonstrated the inhibitory role of the Nb against vascular formation in the chicken CAM (38).

Apparently, Nbs can efficiently block their target antigen in cases where cell killing by Fc mediated effector function is not required. This is probably similar to the activity of Lucentis (Ranibizumab) that also acts in absence of any Fc region (62).

Table 2 gives an overview of studies investigating the effects of Nbs on VEGF, VEGFR, PLGF, HER2/HGF, and EGFR families.

**Table 2:** Examples of published preclinical studies investigating the effect of nanobodies (Nbs) on factors involved in angiogenesis.

Name of selected Nbs	Target	Model or investigated cells	Technique(s) employed	Comments	Results	Reference
Nb22, Nb23, Nb35, and Nb42	Vascular endothelial growth factor (VEGF)	Primary human umbilical vein endothelial cells (HUVECs)	Phage display; cross-reactivity assay; endothelial tube formation assay	Binding affinity from 0.1 to 60 nM; Nbs detect recombinant VEGF-121 and VEGF-165	Inhibition of endothelial cell proliferation or tube formation	(53)

ZFR-5	VEGF	HUVECs	Phage display; whole-cell ELISA experiments; endothelial cell assay	Evaluation of six phage-displayed Nbs from an immune phage library	ZFR-5 blocked interaction of VEGF with its receptor; significant inhibition of proliferation response of HUVECs to VEGF	(50)

V12	VEGF	Chorioallantoic membrane (CAM) of fertilized eggs	Phage display; non-competitive enzyme immunoassay; CAM assay	Twenty-four clones were tested by monoclonal phage ELISA	VA12 Nb showed substantial anti-angiogenesis activity	(51)

3VGR19	VEGF receptor (VEGFR)-2	293KDR and HUVECs	Phage display; fluorescent activated cell sorter (FACS) analysis; endothelial tube formation assay	293KDR cells express high levels of VEGFR-2	Nb recognized antigen on cell surface and inhibited endothelial tube formation	(54)

Nb-C18	Placental growth factor	Chicken CAM model; HUVECs	Phage display; 3D-capillary tube formation assay; transwell migration assay; CAM assay	Twelve clones with strong signals were selected	Nb-C18 significantly inhibited proliferation, migration, and 3D-capillary formation of HUVECs; Nb-C18 inhibits vascular formation	(38)

5F7GGC	HER2	BT474M1 breast carcinoma cells; mice bearing subcutaneous BT474M1 xenografts	Phage display; radio-iodination; binding affinity and internalization assays; paired-label biodistribution		Labeling 5F7GGC with *I-SGMIB targeting HER2 expressing malignancies	(63)

2Rs15d	HER2	CHO cells; LS174T, human HER2^+^ colon carcinoma; SKBR3, BT474, and MDA-MB-435D, human HER2^+^ breast cancer; SKOV3, human HER2^+^ ovarian cancer; xenografts mice model	Phage display; ELISA; binding of Nbs in flow cytometry; surface plasmon resonance; Nb labeling; single-photon-emission computed tomography (SPECT)	2Rs15d selected from a panel of 38 Nbs and labeled for tumor imaging	^99m^Tc-labeled 2Rs15d has suitable properties as HER2 tracer for *in vivo* non-invasive imaging	(64)

1E2 and 6E10	Hepatocyte growth factor (HGF)	U-87 MG, human glioblastoma; Bx-PC3, human prostate carcinoma; A549, human alveolar basal epithelial cell carcinoma	Phage display; fusion with albumin-binding Nb; phosphorylation assay; proliferation assay; biodistribution study; nude mice model	1E2 and 6E10 selected among a panel of 12 Nbs which showed good binding to HGF	1E2-Alb8 and 6E10-Alb8 are candidate for therapy and PET imaging of HGF-expressing tumors	(65)

Epidermal growth factor receptor (EGFR) Nb	EGFR	A431; NIH 3T3; HeLa	Phage display; functional panning; Production of multivalent anti-EGFR nanobodies; FACS; A431 animal model	Selection of antagonistic Nb fragments by ligand-specific elution	Untagged Nbs were used for *in vivo* treatment of tumors	(66)

7C12 and 7D12	EGFR	Mice bearing subcutaneous A431 (EGFR^+^) and R1M (EGFR^-^) xenografts	Nb Labeling; Pinhole SPECT/micro-CT imaging; *ex vivo* analysis		High tumor uptake, low liver uptake, and rapid blood clearance	(67)

8B6	EGFR	A431; human prostate carcinoma cell line DU145; HER14 and NIH3T3; MCF-7	Phage display; ^99m^Tc labeling; SPECT; FACS		Nb with high specificity and selectivity toward EGFR overexpressing cells	(68)

^99m^Tc-7C12	EGFR	A431; ICR/CD1 mice; A431 animal model; megalin-deficient mice	Nb labeling; *in vitro* and *in vivo* stability assay; ^99m^Tc-7C12 uptake and biodistribution study		^99m^Tc-labeled 7C12 Nb accumulates significantly in tumor	(69)

^99m^Tc-7C12	EGFR	A431 expressing truncated growth factor; male athymic nude mice	Nb Labeling; *in vitro* binding assay and specificity of ^99m^Tc-7C12; internalization experiment; *in vivo* tumor uptake of ^99m^Tc-7C12; *in vivo* bioluminescence imaging; Pinhole SPECT/Micro-CT		^99m^Tc-7C12 is a candidate for the early prediction of, and treatment of cancer-expressing EGFR	(70)

## Targeting of Other Potential Angiogenesis Activators

### Leptin

Leptin, encoded by the *obese* gene, plays a critical role in the regulation of body weight. The protein is also known as a potent angiogenic factor involved in tumorigenesis, angiogenesis, and metastasis. Leptin regulates VEGF production in human chondrosarcoma and contributes to tumor-associated angiogenesis (71). The involvement of leptin in cancers such as breast, ovarian, and prostate has been demonstrated and thus the development of mAbs against this target has been initiated. However, it remains uncertain whether anti-leptin antibodies entered the clinic (72). Likewise, McMurphy et al. developed an Nb against the leptin receptor, which causes inhibition of growth of a melanoma tumor in mouse. Local administration of a neutralizing Nb, targeting the leptin receptor, at low doses, and adjacent to the tumor, decreased tumor mass with no effects on body weight or food intake (73).

### Endoglin or CD105

CD105 (Endoglin) is one of the tumor-related angiogenesis factors that is upregulated in tumor tissues and neovascularization. This factor activates transforming growth factor beta (TGF-β). Endoglin is a membrane glycoprotein that is a part of the TGF-β receptor complex. Playing a role in tumor angiogenesis, endoglin can be used in diagnosis, prognosis, and therapy (74). The result of phase I of TRC105, a chimeric anti-endoglin (CD105) mAb, in metastatic castration-resistant prostate cancer shows good anti-angiogenic activity and that it is well tolerated (75).

Ahmadvand et al. developed an Nb against CD105, which could inhibit the proliferation of HUVEC cells and tube formation (76, 77).

### HGF and c-Met

Hepatocyte growth factor exclusively induces the growth of endothelial cells without replication of vascular smooth muscle cells and acts as a survival factor against endothelial cell death. In tumor tissues, HGF is a key growth factor linked to increasing cancer progression and angiogenesis. The binding of HGF to its receptor (c-Met, belonging to tyrosine kinase receptors) activates the signaling pathway that causes enhancement of angiogenesis in tumor tissues and prevents apoptosis, which all contribute to the outgrowth of tumors (78). Rilotumumab is an intact mAb that binds to HGF and prevents its association with the c-Met receptor. This mAb is currently in clinical phase trials for treatment of different solid tumors (79). Likewise, two Nbs against HGF, referred to as 1E2 and 6E10, were identified and modified for serum half-life extension by fusion with an albumin-binding Nb (Alb8). 1E2-Alb8 and 6E10-Alb8 Nbs binding to HGF inhibits recognition of the c-Met receptor. After labeling with ^89^Zr, a positron emitter, the biodistribution of Nbs was evaluated in nude mice. The result of these animal studies revealed a tumor growth inhibitory effect of these Nbs in a glioblastoma xenograft model (65).

It is well established that the activation of the c-Met receptor by HGF and its subsequent signaling and angiogenesis activation is involved in many human malignancies (80). Interfering with the signaling by ligand or adding c-Met dimerization blocking antibodies or kinase inhibitors all exert a measurable inhibitory effect on cancer cell progression (81). Onartuzumab, H224G11/ABT700, LY2875358, and ARGX-111 are mAbs against c-Met in clinical trials that are exploiting exactly this therapeutic strategy (82). Slordahl et al. developed an anti c-Met Nb that effectively prevented thymidine incorporation by ANBL-6 MM cells *via* inhibition of an HGF autocrine growth loop and thymidine incorporation into INA-6 MM cells induced by exogenous HGF. Migration and adhesion of INA-6 was completely and specifically abolished by the Nb. Apparently, the Nb also reduces the inhibitory effect of HGF on bone morphogenetic protein-2-induced alkaline phosphatase activity and the mineralization of human mesenchymal stem cells (83). Finally, Heukers et al. developed an Nb delivery system constructed from their anti c-Met Nb decorated albumin nanoparticles (anti-Met-NANAPs). Targeting of c-Met expressing cells could downregulate the HGF receptor protein (84).

### Epidermal Growth Factor Receptor

The EGFR belongs to the HER/ErbB family of receptor tyrosine kinases (85). This family includes HER-1 (EGFR/ErbB-1), HER2 (neu, ErbB-2), HER-3 (ErbB-3), and HER-4 (ErbB-4) (86). Overexpression of EGFR may confer or promote a malignant phenotype and increase the tumor mass (87). One of the processes to enhance tumor angiogenesis consists in the activation of the EGFR pathways (66). Increased levels of EGF and TGF-β can cause activation of VEGF and subsequent tumor angiogenesis (88). On the basis of the pro-angiogenic properties of EGFR, blocking EGFR downregulates VEGF, IL-8, and basic FGF production, interrupts upstream angiogenesis signaling pathways, and is accompanied by a net reduction in microvessel density and metastases (88).

Roovers et al. (89) retrieved the first antagonistic anti-EGFR Nbs for cancer therapy by competitively eluting the EGFR-attached Nbs with EGF. This strategy resulted in the selection of a panel of Nbs that inhibited the recognition of EGF to its receptor without acting as a receptor agonist. The results confirmed that these Nbs inhibited perfectly EGF-induced signaling and EGF-induced cell proliferation *in vitro* and prevented the tumor outgrowth in animal models. Tijink et al. targeted tumors using a bivalent anti-EGFR Nb (αEGFR− αEGFR) that was also fused to a Nb against albumin to improve the biodistribution and circulation time of the construct. To facilitate their quantification, the proteins were radiolabeled with ^177^Lu. Tumor uptake of ^177^Lu-αEGFR-αEGFR-αAlb decreased from 5.0 ± 1.4 to 1.1 ± 0.1 %ID/g between 6 and 72 h after injection. Remarkably, this multi-modal construct not only decreased blood clearance but also increased penetration to tumor tissue (22). In 2011, the group of Roovers in continuation of their previous work improved the potential of EGFR Nbs by combining Nbs with specificities similar to both Cetuximab and Matuzumab into a single bi-paratopic molecule (90). This bi-paratopic construct could bind simultaneously to two independent epitopes that overlap with those of Cetuximab and Matuzumab. This Nb (referred to as CONAN-1) could inhibit the cell proliferation that depended on EGF *in vitro* and it could also inhibit the tumor outgrowth with an almost similar potency as the entire Cetuximab mAb and it was more potent than the bivalent, mono-specific Nbs (90). In a recent independent study, Farasat et al. improved the affinity of an EGFR Nb (7D12) by *in silico* tools (91).

Human EGFR 2 (HER2) or ErbB-2 is another member of EGFR family. Uncontrolled expression of HER2 occurs in about 20–30% of breast cancers, 4–6% of non-small cell lung cancers, 20–24% of gastric cancers, and also in colon and ovarian cancers (92). Trastuzumab (Herceptin^®^, Genentech) is the approved humanized and intact IgG1 mAb, which in combination with Pertuzumab has been used to treat patients with HER2 positive malignancies (93, 94).

Patients with a strong positive result of their biopsy in immunohistochemistry or with the gene amplification assay are expected to benefit from a Trustuzumab therapy. Moreover, with the available HER2 targeting Nbs and fast clearance of excess administered Nb, it became apparent how useful Nbs might be for non-invasive imaging and to screen *in vivo* for HER2^+^ carcinomas (13). Obviously, if Nbs are shown to be excellent tumor targeting molecules, then substitution of the nuclide into a more toxic payload will produce a potent compound for targeted radionuclide therapy. Thus, the HER2 targeting Nbs were used for imaging and radiotherapy strategies (95). There are several criteria that dictate the choice for employing radiolabeled nuclides. The radiolabeled prosthetic group should have the least possible toxicity in healthy tissues, high tumor penetration, prolonged residence time at the tumor tissue site and fast clearance of the excess drug from normal tissues, and low retention time in the kidneys and other non-targeted organs. Obviously, the selected nuclide conjugated to the Nb should not change the binding properties to its cognate target (12, 96). According to different studies, a carefully selected anti-HER2 Nb is one of the best candidates to be used as tracer in different imaging strategies such as PET (positron emission topography) or single-photon-emission computed tomography (SPECT) imaging. Vaneycken et al. investigated the performance of about 40 Nbs against HER2 and identified the lead Nb to trace xenografted tumors in mice (64). Their 2Rs15d Nb, which does not compete for HER2 binding with Traztuzumab or Pertuzumab, was selected based on its microbial expression level, SPR-measured affinity, cell targeting in flow cytometry, and radio-ligand binding studies. The use of ^99m^Tc-labeled 2Rs15d in SPECT imaging quantification and biodistribution analyses demonstrated high tumor uptake in two HER2^+^ tumor models, fast blood clearance of excess Nb, and low accumulation in non-target organs except kidneys.

Xavier et al. (97) constructed ^68^GA-NOTA (1,4,7-triazacyclononane-1,4,7-triacetic acid) anti HER2 Nbs for a dosimetry and toxicity assay in interim PET imaging. The biodistribution studies showed fast and specific uptake in HER2 positive tumors, resulting in high-contrast PET/CT images. High-specific contrast imaging and lack of toxicity of ^68^Ga-NOTA-2Rs15d are ideal properties for human clinical trials with PET tracers. Interestingly, D’Huyvetter et al. observed that the kidney retention of labeled Nb 2Rs15d was reduced significantly upon removal of the haemaglutinin tag and the His tag, in line with the notion that the kidney retention is dominated by the amino acid composition in these C-terminal tags (98).

Recently, Keyaerts et al. reported the results of a phase I study of ^68^Ga-HER2 Nb in a PET/CT assessment of HER2 expressing breast carcinoma. Their data showed that this Nb has a favorable biodistribution and is safe to use and well tolerated in patients. Acceptable signals in the kidneys, liver, lacrimal glands, and intestines were noted and very low background levels in other organs were observed. Therefore, it seems that this Nb used as a molecular probe might replace histochemical analysis of biopsies in the future (13).

The very low uptake in other organs except the targeted tumor of Nbs in the absence of any tag is considered to be safe for human administration and effective for targeted radionuclide therapy. Indeed, Nb-based targeted radionuclide therapy led to an almost complete blockade of tumor growth in xenograft model. Thus, a very promising strategy was developed by D’Huyvetter et al. (98). They also replaced the ^99m^Tc with ^177^Lu on the HER2 Nb using a bifunctional chelator for better *in vivo* behavior and optimized radiolabeling. This adaptation had no effect on the tumor targeting capacity of the Nb in xenografted mice, and *ex vivo* biodistribution studies showed a significant quantity of the therapeutic Nb on tumors expressing medium HER2 levels and low background activity in other tissues except for the kidneys (99).

In an alternative therapeutic approach, Van de Broek et al. (100) conjugated the 2RS15d onto branched gold nanoparticles for photothermal therapy. It is hypothesized that laser irradiation at the site of nanogold accumulation will produce enough heat to destroy tumor cells overexpressing HER2 receptor in contrast to control cells (100). Unfortunately, the biofunctionalized branched gold particles seem to suffer from a serum albumin corona when used *in vivo*. However, this has recently been remediated by preparing the nanoparticles using a different blocking agent to inactivate the reactive groups (101).

Finally, 5F7GGC, originally introduced as an HER2 Nb (89), was radio-iodinated using a conventional method (Iodogen) and IB-Mal-D-GEEEK reagent (102). The radio-iodinated 5F7GGC Nb using the^131^I-IB-Mal-D-GEEEK indicated better tumor blocking properties both *in vitro* and *in vivo* compared with Nb labeled *via* Iodogen. Moreover, the toxicity of ^131^I-IB-Mal-D-GEEEK for healthy tissue is lower than that of tumors except in the kidneys where substantially higher radioactivity levels were observed. Radio-iodinated 5F7GGC Nb with the residualizing agent *N*-succinimidyl 4-guanidinomethyl 3-^125^/^131^I-iodobenzoate (*I-SGMIB) not only presented a promising new conjugate for targeting HER2-expressing malignancies but also showed improved tumor retention and faster normal-tissue clearance than ^131^I-IB-Mal-D-GEEEK (63). However, further investigation is needed to demonstrate the potential utility of *I-SGMIB-5F7GGC labeled with ^124^I, ^123^I, and ^131^I for PET and SPECT imaging and for targeted radiotherapy.

Likewise, Jamnani et al. developed oligoclonal Nbs against HER2 receptor by selecting Nbs against the native receptor exposed on the cell surface. These oligoclonal Nbs inhibited growth of breast cancer cells better than each individual Nb (103). Next, genetically engineered T cells were generated expressing chimeric antigen receptors (CAR) comprising five individual HER2 specific Nbs joined to various CD28 and OX40 signaling endodomains. The use of these oligoclonal anti-HER2 Nbs-CAR engineered T cells in an adoptive cell therapy resulted in higher cytokine secretion and enhanced cytotoxicity against HER2^+^ tumor cells (104).

The application of mAbs in radio-immunotherapy has some severe limitations such as a poor tumor penetration due to their large size and undesirable pharmacokinetics. To date, only two radiolabeled mAbs have been approved for commercial use, ^90^Y-Ibritumomab (Zevalin, Biogen-Idec Pharmaceuticals) and ^131^I-Tositumomab (BEXXAR, GlaxoSmithKline), both of which have been used to treat indolent B-cell lymphoma (105, 106).

Table 2 gives an overview of studies investigating the effects of Nbs targeting EGFR and HER2.

### Chemokine Receptor Type 7 (CXCR7)

Chemokine receptor type 7 is one of the members of the chemokine receptor family, belonging to the superfamily of G protein-coupled receptors. The overexpression of chemokines and chemokine receptors in various tumor types and their involvement in cell proliferation, metastasis, and angiogenesis has opened new avenues in targeting the chemokine receptors (107). Maussang et al. reported the development an anti-CXCR7 Nb to reduce head and neck cancer cell growth *in vivo*. Apparently, these Nbs act by inhibiting angiogenesis. The interference with the pathway led to the inhibition of β-arrestin-2 signaling and secretion of angiogenic chemokine ligand 1 (108).

Table 2 gives an overview of studies investigating the effects of Nbs on the major factors known to be involved in the angiogenesis process.

## Conclusion and Future Prospects

Although there are a large number of angiogenesis inhibitors, remaining clinical problems, including resistance from the tumor microenvironment, enhanced tumor hypoxia, and reduced delivery of chemotherapeutic agents, have curtailed their full therapeutic potential. Given that mAbs have shown considerable success in tumor-targeted therapies during the past couple of decades, an increasing focus is now going toward remediation of the therapeutic limitations of antibodies, such as those related to their large and complex structure. The introduction of Nbs, a single-domain antigen-binding fragment has demonstrated that they can overcome some drawbacks of intact antibody-based cancer therapeutics and diagnostics. The Nb seems to become a promising therapeutic agent for cancers. Their angiogenesis inhibiting potential can be employed in various ways—as receptor antagonist, by targeting effective epitopes, and by decorating nano-carrier surfaces (17, 109). The small size of Nbs is highly suitable for generating tracers for non-invasive *in vivo* diagnosis of malignant cells. In targeting angiogenesis, Nbs should be equipped with a half-life extension moiety to increase the tumor loading capacity. The recognition of distinct epitopes that is often inaccessible for classical antigen-binding fragments might also broaden their therapeutic applications. Despite the challenges related to the efficacy of Nbs resulting from the lack of natural effector functions as present in the Fc region, the Nb holds promises, both for diagnosis and therapy of cancer.

## Author Contributions

All authors listed have made a substantial intellectual contribution to write the text and have approved it for publication.

## Conflict of Interest Statement

The authors declare that the research was conducted in the absence of any commercial or financial relationships that could be construed as a potential conflict of interest. The reviewer JT and handling editor declared their shared affiliation.
